# Progress in Translating Glaucoma Genetics Into the Clinic: A Review

**DOI:** 10.1111/ceo.14500

**Published:** 2025-02-10

**Authors:** Antonia Kolovos, Giorgina Maxwell, Emmanuelle Souzeau, Jamie E. Craig

**Affiliations:** ^1^ Flinders Health and Medical Research Institution Flinders University Adelaide Australia; ^2^ Department of Ophthalmology Flinders Medical Centre Adelaide Australia

**Keywords:** glaucoma, mendelian, monogenic, polygenic risk scores, polygenic scores

## Abstract

Precision medicine is paving the way for personalised risk assessment, and its translation into glaucoma clinics holds potential to change current management paradigms. Our understanding of glaucoma's genetic architecture has expanded in recent years, recognising both monogenic and polygenic contributions. Genetic testing within glaucoma populations can provide additional information for clinicians to support decision‐making. Here, we review the evidence base for genetic variants strongly associated with glaucoma and outline a vision for translating these learnings into the clinic. Integrating clinical and genetic information will provide clinicians and patients with the strongest evidence to deliver personalised glaucoma management.

## 1 Introduction

Glaucoma is a multifarious disease encompassing several disorders culminating in a progressive optic neuropathy and vision loss. Despite recent diagnostic and therapeutic advancements, glaucoma remains the leading cause of irreversible blindness globally [1, 2]. This heterogenous disease affects individuals in all stages of life, from congenital to adult‐onset forms. Glaucoma is primarily heritable with both monogenic and polygenic components. This review will consider genetic testing in a clinical setting for adult open‐angle glaucoma (OAG), juvenile OAG (JOAG), primary congenital glaucoma (PCG) and anterior segment dysgenesis (ASD) defined as follows. PCG is characterised by angle anomalies, often with buphthalmos [3]. JOAG and OAG are characterised by normal angles, with JOAG defined by an age at diagnosis between 4 and 30 to 40 years, after which glaucoma is classified as adult‐onset OAG [3]. PCG and JOAG develop in the absence of acquired or non‐acquired conditions, whilst ASD is defined as the non‐acquired anomalies of the iris, lens and/or cornea [3].

The purposes of genetic testing may differ based on the glaucoma subtype and gene/s investigated. For monogenic (single gene) glaucoma, genetic testing could offer information about diagnosis, prognosis and management and hold implications for family members. For polygenic (multiple genes) glaucoma, which has been associated with adult‐onset OAG, genetic testing may offer additional risk information to guide screening, surveillance and interventions. In this review, we build a case for clinical genetic testing in glaucoma management. We share cases where genetic testing has informed clinical management. We outline the current strengths and limitations of existing genetic testing and propose an integrated model for the clinic setting.

## 2 Genetic Determinants of Glaucoma

There is a strong genetic contribution to the risk of developing glaucoma. The genetic heritability of primary OAG is estimated at 70%, making it one of the most heritable common complex diseases, more than cardiovascular disease and cancer [4]. There is a broad spectrum of genetic variations associated with glaucoma risk, including both monogenic and polygenic risk factors.

Monogenic (or Mendelian) conditions are primarily driven by genetic variants of high effect size in a single gene. Genetic variants associated with monogenic glaucoma are typically less frequent in the population and are predicted to be deleterious to the associated gene, subsequently conferring an increased risk of developing the disease. Mendelian glaucoma‐associated genetic variants have been shown to account for around 3%–4% of overall adult‐onset glaucoma and higher rates of younger age of onset glaucoma [5, 6].

The vast majority of OAG cases are not explained by a single monogenic variant, even when familial aggregation of disease is present [7]. Instead, the genetic architecture of adult‐onset OAG is believed to be primarily polygenic, with hundreds to thousands of common genetic risk variants. Most variants are typically common, defined as having a minor allele frequency of 5% or more, in the reference population, unlike variants of monogenic glaucoma, which are typically rarer. Each common variant may only have a relatively small impact on glaucoma risk, either protective or deleterious; however, when aggregated together, they confer a significant risk. This is illustrated as a polygenic risk score (PRS, which may be referred to as a genetic risk score (GRS) or polygenic score (PGS)). A PRS represents the aggregate risk conferred by many common genetic risk variants associated with a disease or trait. It may be expressed as a raw or normalised score adjusted to scores from an appropriate reference population (e.g., ancestrally matched individuals from the 1000 Genomes cohort). For example, an individual with a normalised glaucoma PRS in the 95th percentile represents someone in the top 5% of polygenic risk based on the PRS distribution of the reference population [8]. Importantly, a PRS is not diagnostic, but an estimate of risk. Individuals with very high PRS for glaucoma have risk at least as great as that conferred by monogenic variants and account for a vastly greater number of adult onset OAG cases than Mendelian forms [8].

**BOX 1: Key messages**

1. PRSs are a measure of genetic susceptibility towards a trait or disease. They represent the aggregate risk conferred by many common genetic risk variants associated with the disease.
2. Genetic variants included in a PRS are ‘common’, defined as having a minor allele frequency of 5% or more in a reference population. This is in contrast to monogenic (or Mendelian) glaucoma, which is usually associated with ‘rare’ variants with very low population frequencies.
3. A PRS is not diagnostic, but an estimate of risk, and can be used as a decision‐support tool.

### 2.1 Monogenic Glaucoma

Monogenic causes occur across the glaucoma spectrum, from primary to secondary and from congenital to adult‐onset. They are more prevalent in individuals with a younger age of onset. The rate of genetic diagnosis depends on a number of factors, including the definition, classification and inclusion of glaucoma subtypes, the population studied, the genetic testing approach, and result interpretation strategies. The majority of studies have only sequenced known glaucoma genes in specific cohorts. A few studies have applied exome/genome sequencing or targeted gene panels to assess genetic diagnosis rates in cohorts with specific subtypes of glaucoma, including PCG [9, 10], ASD [11, 12], childhood glaucoma [13, 14, 15] and POAG [16, 17, 18]. However, there is a paucity of data regarding genetic diagnoses across the entire glaucoma spectrum.

**FIGURE 1:**
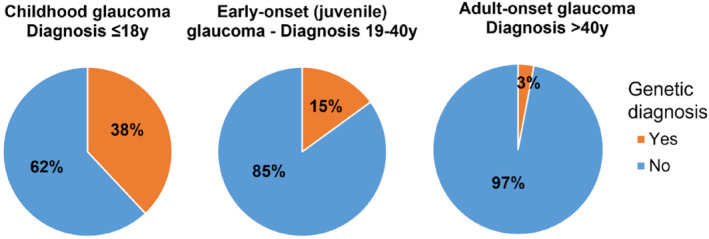
Diagnostic yield across the glaucoma spectrum.

The Australian and New Zealand Registry of Advanced Glaucoma (ANZRAG) is the world's largest repository of genetic samples from individuals with severe vision loss from glaucoma and their family members [19]. It comprises > 9400 participants, including 5490 participants with glaucoma, 3898 participants with adult‐onset POAG, 397 participants with early‐onset POAG (diagnosed < 40 years) and 379 participants with childhood glaucoma (diagnosed < 18 years; accessed 20th September 2024). The genetic testing programme of the ANZRAG includes targeted screening for adult‐onset glaucoma, as well as targeted screening and exome sequencing for childhood and early‐onset glaucoma. Recent data identified a Mendelian genetic diagnosis in 38% of childhood glaucoma, 15% of early‐onset POAG [20] and 3% in adult‐onset POAG (Figure 1).

Monogenic glaucoma displays genetic heterogeneity (more than one gene causes the same glaucoma subtype) and phenotypic heterogeneity (the same gene can lead to multiple phenotypes). Genetic variants can be associated with incomplete penetrance (not all individuals with pathogenic variants will express the associated phenotype), age‐related penetrance (the prevalence of those individuals with pathogenic variants expressing the phenotype increases with age) and variable expressivity (pathogenic variants can lead to a range of symptoms or signs across a phenotypic spectrum). These aspects contribute to a broad phenotypic spectrum and overlap between clinical entities and lead to challenges for genetic testing and counselling. Digenic causes of glaucoma have been reported [21, 22]; however, there is limited evidence to support the pathogenicity of the different genetic variants identified. Whilst it is highly plausible that additive effects of rare variants could have a role in disease causation, evidence is currently lacking.

**FIGURE 2:**
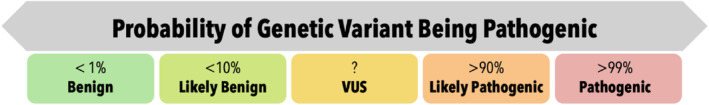
Visual representation of the classification system by ACMG and AMP for the pathogenicity of Mendelian variants. VUS, variant of uncertain significance.

The pathogenicity of Mendelian variants is established by clinical genetic testing laboratories using guidelines developed by the American College of Medical Genetics and Genomics (ACMG) and the Association for Medical Pathology (AMP) [23]. Variants are assessed against criteria reflective of different types of evidence (e.g., population, computational, functional, segregation data), which can be applied at different strengths of evidence. The criteria applied for and against pathogenicity are then combined to lead to a five‐level probability‐based classification system, as demonstrated in Figure 2.

Initiatives that periodically review and update gene–disease associations (e.g., ClinGen Gene Curation Expert Panels [24], PanelApp [25]), and review evidence for variant classification with FDA approval such as the ClinGen Glaucoma Variant Curation Expert Panel [26], are essential in improving the validity and accuracy of genetic variants.

The different genes associated with glaucoma, their inheritance and phenotypic spectrum are detailed in Table 1, although a full description of all associated genes is beyond the scope of this article. The most common Mendelian gene associated with adult‐onset glaucoma is *Myocilin* (*MYOC*) [29]. Variants in *MYOC* are usually associated with strong family history, high IOP, variable age of onset, and more severe glaucoma if untreated [5, 29]. The most common *MYOC* variant (p.Gln368Ter) has a mean age at diagnosis of 52 years with incomplete penetrance, whilst other variants such as p.Pro370Leu or p.Gly367Arg can lead to glaucoma in childhood or early adulthood [5, 46]. Other monogenic causes of normal‐tension adult‐onset glaucoma include the p.Glu50Lys variant in *Optineurin* (*OPTN*), *METTL23* [47] and copy number variations (duplications or triplications) of *TBK1* [31, 48]. These four genes exhibit an autosomal dominant pattern of inheritance.

**TABLE 1:** Genes associated with isolated glaucoma or glaucoma associated with non‐acquired ocular anomalies.

Gene	Inheritance	Associated glaucoma types
*MYOC*	AD	JOAG (8%–36%) [27, 28], POAG (2%–4%) [29]
*OPTN*	AD	POAG (rare) [30]
*TBK1*	AD	POAG (rare) [31]
*CYP1B1*	AR	PCG (15%–100%) [32, 33], JOAG (13%) [20]
*TEK*	AD	PCG (5%), JOAG (rare) [34]
*ANGPT1*	AD	PCG (rare) [35]
*THBS1*	AD	PCG (rare) [36]
*EFEMP1*	AD	JOAG (rare) [37]
*FOXC1*	AD	ARS [38]
*PITX2*	AD	ARS [38]
*PAX6*	AD	Aniridia (90%) [39]
*TRIM44*	AD	Aniridia (rare) [40]
*PXDN*	AR	ASD (rare) [41]
*LTBP2*	AR	ASD (rare) [42]
*CPAMD8*	AR	ASD (rare) [43]
*FOXE3*	AD/AR	ASD (rare) [44]
*PITX3*	AD	ASD (rare) [45]

Abbreviations: AD, autosomal dominant; AR, autosomal recessive; ARS, Axenfeld–Rieger syndrome; ASD, anterior segment dysgenesis; JOAG, juvenile open‐angle glaucoma; N/R, not reported; PCG, primary congenital glaucoma; POAG, primary open‐angle glaucoma.

The two main contributors of JOAG are *MYOC* and *CYP1B1*. Because *MYOC* variants have an autosomal dominant inheritance and *CYP1B1* variants have an autosomal recessive inheritance, a multi‐generation family history of glaucoma could support the presence of *MYOC* variants, whilst an absence of family history could suggest *CYP1B1* variants. However, *de novo* variants have rarely been reported in *MYOC* in the absence of affected relatives [31] and some individuals may not have a family history or may not be aware of it (15% of probands with *MYOC* variants in the ANZRAG reported no known family history). Therefore, the absence of family history should not necessarily rule out the possibility of dominantly inherited variants.

Variants in *CYP1B1* are the most common cause of PCG. They account for 15%–20% of cases in Asian and European populations [32, 49, 50] and as high as 90%–100% in Middle Eastern and Romani populations, which exhibit higher rates of consanguinity and strong founder effects, respectively [33, 51]. More recently, variants in *TEK* have been implicated in 5% of individuals with PCG [34], and variants in *FOXC1* have been reported in 5% individuals with suspected PCG but with many later reclassified as having Axenfeld–Rieger syndrome (ARS) [52].

ASD is a heterogenous group of conditions comprising ARS and aniridia amongst the most common monogenic phenotypes. ARS is explained by genetic variants and copy number variations (deletions and duplications) of *FOXC1* and *PITX2* in 40%–70% of individuals [38, 53]. In contrast, over 90% of aniridia are caused by variants and deletions involving *PAX6* or regulatory elements of *PAX6* [39, 54].

### 2.2 Polygenic Glaucoma

Recent years have established a strong polygenic contribution to adult‐onset OAG disease. Genome‐wide association studies (GWAS) are genetic studies that identify common genetic risk variants, known as single nucleotide polymorphisms (SNPs), associated with a disease or trait. Hundreds of SNPs have been identified in association with OAG, intraocular pressure (IOP) and vertical cup‐to‐disc ratio (VCDR) [55]. IOP and VCDR are two highly heritable traits, termed endophenotypes [56, 57], that confer glaucoma risk.

The genetic power of endophenotypes can be leveraged through a method known as multi‐trait analysis of GWAS (MTAG) [58]. In this combined approach, many of the strongly associated SNPs are not significantly associated individually with an input trait but reach the threshold for significant association with glaucoma due to the MTAG method of leveraging the clinical correlation between traits (Table S1). This increases the explained heritability of OAG, and the predictive power of the PRS generated from these GWAS data. This methodology has enabled substantial increases in the effective sample size available for glaucoma gene discovery.

The first comprehensive PRS for OAG was built from an MTAG using GWAS summary data for OAG and its endophenotypes IOP and VCDR [59]. The MTAG study identified 114 independent risk variants from 107 loci in the discovery cohort, including 49 previously undiscovered loci. This translated to a PRS comprising 2673 uncorrelated risk variants [59], not all of which were genome‐wide significant hits in the GWAS but which nonetheless provided added power in predicting glaucoma diagnosis. Supporting Information Table outlines the top 100 SNPs tested for in this multi‐trait OAG PRS and their strength of association to the trait.

Expanding on this work, larger‐scale meta‐analyses of multiple glaucoma GWAS have identified additional risk variants, increasing the explained heritability of glaucoma from 9.4% to 14.9% [60, 61]. These more recent studies have included multi‐ancestry approaches, thus starting to address a limitation of previous work.

The clinical validity of a glaucoma PRS has been demonstrated in different populations. A higher glaucoma PRS has been associated with greater odds of developing glaucoma [59, 62, 63, 64, 65, 66], earlier age at diagnosis [59, 65, 67], more affected family members [59, 67], higher peak IOP [67], a need for more rapid escalation of treatment [68], greater risk of vision loss despite treatment [69] and increased risk of incisional surgery [66, 70].

### 2.3 Combining Monogenic, Polygenic and Clinical Risk Features

The penetrance of the most common disease‐causing *MYOC* variant p.Gln368Ter is incomplete and is lower in population‐based than family‐based studies [5, 29, 71]. A glaucoma PRS has been demonstrated to modify the penetrance of this variant within the UK Biobank [59, 72]. Individuals carrying *MYOC* p.Gln368Ter in the highest PRS tertile compared to the lowest tertile had a sixfold increased risk of glaucoma diagnosis and a younger age of diagnosis [59]. There is currently no data on how PRS modifies the penetrance of other *MYOC* variants or other Mendelian OAG genes (e.g., *TBK1*, *OPTN* and *CYP1B1*) due to their low population frequency.

Genetic testing for PRS and rare variants are currently processed separately due to using different testing methodologies. Imputation (statistically inferring unobserved genotypes) of rare variants like *MYOC* p.Gln368Ter is possible [73] but accuracy is indirectly proportional to allele frequency; thus, rare variants are often excluded. Newer reference panels incorporate over 7 million SNPs [74], offering improved imputation accuracy and increased genetic power. However, monogenic glaucoma can include rare variants or other mechanisms (e.g., copy number variations) that would not be detected by the arrays commonly used for PRS calculation. This means that monogenic variants are currently not assessed when performing polygenic testing. The increasing affordability of technologies such as whole genome sequencing or targeted sequencing may provide a more complete picture in genetic testing in the near future.

Glaucoma risk prediction models are significantly improved by integrating demographic and/or clinical risk factors with polygenic risk [64, 75, 76, 77]. For example, the area under the curve (AUC) of predictive models increased from 0.72 (PRS alone) to 0.79 when demographic predictors were included (PRS + sex + age) and to 0.89 when clinical risk factors were included (PRS + sex + age + IOP + VCDR) [59, 62]. PRS improves the capture of inherited genetic risk compared to self‐reported family history, which is prone to bias and inaccuracy (family history + age + sex, AUC 0.73; versus family history + age + sex + PRS, AUC 0.80) [58, 78]. Similarly PRS alone provides a risk similar to family history with risk factors (PRS, AUC 0.75; versus age + sex + IOP + family history, AUC 0.75) [62]. Models may also integrate other glaucoma risk factors including corneal biomechanics, IOP fluctuation, cardiovascular risk factors and retinal imaging (e.g., thickness of the retinal nerve fibre layer and the ganglion cell complex). Prediction models have previously been developed based on existing clinical risk factors [79]. New risk prediction tools that integrate genetic, clinical and demographic factors with application across a broad range of indications will need to be developed for effective implementation of PRS into clinical practice.

## 3 Integrating Genetic Testing Into the Clinic

Glaucoma remains a clinical diagnosis. However, by integrating genetic testing into the clinic, diagnosis may be made earlier in the disease course, treated more proactively and monitored more appropriately, all of which will reduce the risk of severe visual field loss. In this section, the authors aimed to provide a contextual overview of the benefits and clinical applicability of genetic testing for glaucoma, illustrated with case examples.

### 3.1 Genetic Testing: Indications and Purpose

Genetic testing for eye conditions that are primarily Mendelian is generally supported by recommendations [80]. This includes offering genetic testing for childhood glaucoma, which demonstrates clinical utility, including altered surveillance and/or management [81, 82]. In comparison, genetic testing of rare variants for complex conditions such as adult‐onset OAG is currently not supported [80]. Whole population screening for glaucoma by clinical examination is not currently recommended due to lack of evidence for cost‐effectiveness in achieving better outcomes [83, 84]. It should be noted these recommendations did not consider polygenic testing. However, polygenic testing at a population‐level or in a targeted population may be supported with clear evidence for improved health outcomes in a cost‐effective manner.

There are different benefits to offering genetic testing for glaucoma, including improving monitoring, management and prognosis and providing information to families on at‐risk relatives and reproductive options. The indication and support for testing vary depending on the type of glaucoma, age of onset and purpose of testing. The different types of genetic testing are listed in Table 2, with case examples described below.

**TABLE 2:** Types and aims of genetic testing.

Type of testing	Aim of testing
Diagnostic	Genetic testing for an affected individual to establish a genetic diagnosis (e.g., testing for childhood glaucoma‐associated genes in an affected individual). Can provide clarity on a diagnosis, guiding treatment and management decisions.
Predictive/presymptomatic	Genetic testing for unaffected/asymptomatic relatives of individuals who have received a genetic diagnosis (e.g., cascade testing for relatives of an individual with a *MYOC* variant). Can provide information about the risk of developing a condition.
Screening	Genetic testing for unaffected individuals to identify those at risk of developing a condition (e.g., polygenic risk testing in an unaffected individual).
Carrier	Genetic testing for relatives of an individual with variants associated with an autosomal or X‐linked recessive inheritance or through reproductive genetic carrier screening (e.g., testing parents of an individual with *CYP1B1* variants). Can provide information about the likelihood of having an affected pregnancy/child.
Segregation	Genetic testing for relatives of an individual with multiple variants, or variants of uncertain significance, to inform their pathogenicity and causality with phenotype, or in autosomal recessive forms to confirm the parents are carriers and the variants are on different alleles.
Prenatal	Genetic testing for an embryo/foetus either because of carrier status of the parents or because of an established genetic diagnosis in the family and when there is an established risk that the pregnancy can be affected. This type of testing is generally done via chorionic villus sampling, amniocentesis or non‐invasive prenatal testing.
Preimplantation testing for monogenic conditions (PGT‐M)	Genetic testing for a fertilised embryo either because of carrier status of the parents or because of an established genetic diagnosis in the family and when there is an established risk that the pregnancy can be affected to greatly reduce the chance of having a child with the condition screened for. This type of testing involves designing a custom test for the biological contributors to the pregnancy and the specific genetic change/s to identify and select unaffected embryos for transfer/implantation.

### 3.2 Monogenic Testing

Glaucoma management is guided by clinical presentation and features; therefore, genetic testing is not essential for diagnosis or management. However, genetic results may inform the choice or timing of interventions depending on patients' glaucoma presentation and treatment compliance if they are known to be associated with more severe outcomes. For example, a recent study showed the potential benefit of gonioscopy‐assisted transluminal trabeculotomy in treating *MYOC*‐associated JOAG [85]. Similarly, establishing a genetic diagnosis is crucial for timely access to clinical trials for conditions with gene‐specific therapies such as for inherited retinal diseases. Whilst gene‐specific therapies currently do not exist for glaucoma, some studies have shown promising results to improve the glaucoma phenotype in *MYOC* mice models using either chemical chaperones (phenylbutyrate) or CRISPR genome editing [86, 87].

There are additional clinical scenarios where diagnostic genetic testing could benefit individuals with glaucoma. Data from the ANZRAG recently showed that genetic testing led to a change in clinical diagnosis in 10% of individuals with childhood or early‐onset glaucoma, which may inform management of ocular and potential systemic features [20]. Additionally, genetic testing can lead to the diagnosis of unsuspected syndromes associated with glaucoma (Case 1). This may be because of an absence or unknown family history, or systemic features that have not yet developed are subtle or unrecognised. This highlights the importance of obtaining a thorough medical and family history to identify potential monogenic and syndromic forms of glaucoma.

Case  1 Syndromic glaucoma.

**FIGURE 3:**
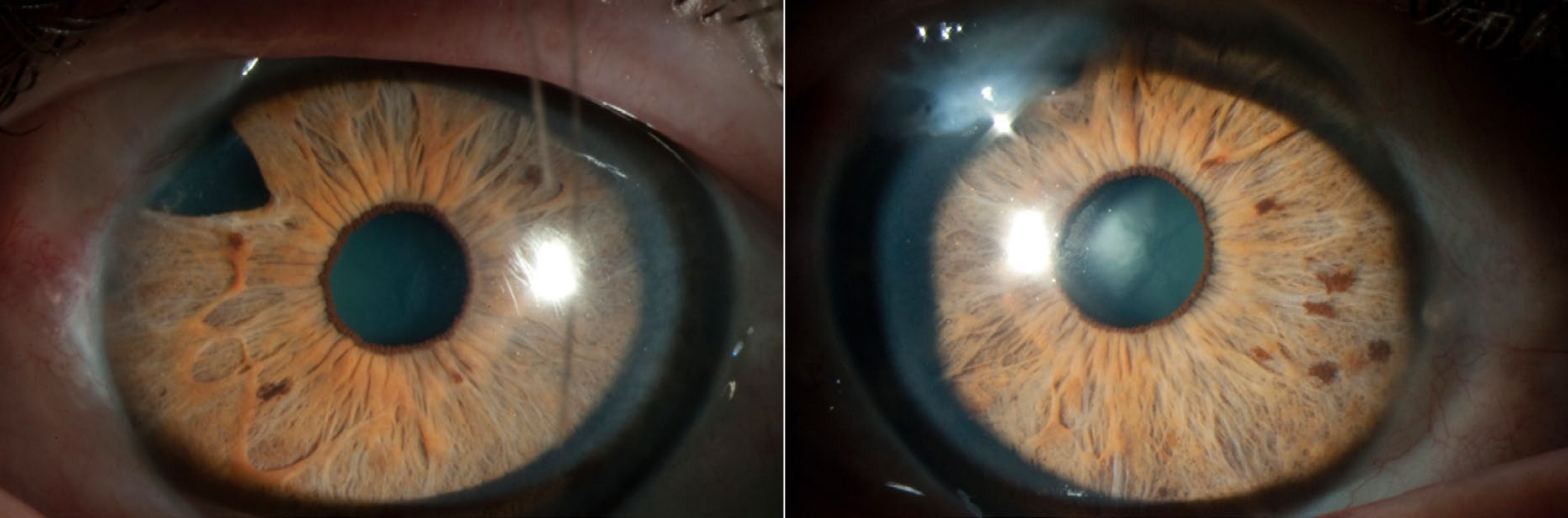
Bilateral photographs of the proband from Case 1 with a *PITX2* pathogenic variant, illustrating reduced stromal features. Evidence of trabeculectomy bilaterally.

A woman was diagnosed with glaucoma at 25 years. She had mild iris stromal hypoplasia but no other signs of anterior segment dysgenesis (Figure 3). She had a positive family history of glaucoma with her father and paternal grandmother affected and a sibling with ocular hypertension. When she was 48 years old, genetic testing identified a pathogenic variant in *PITX2*, associated with ARS. Upon re‐examination, she had mild peripheral anterior synechiae, dental malocclusion and an umbilical hernia, all consistent with ARS. Genetic testing of family members identified the same *PITX2* variant in her father, sibling and an unaffected daughter. Her sibling and daughter had iris stromal hypoplasia, microdontia and redundant periumbilical skin. Although signs of anterior segment dysgenesis were subtle in this family, the family and medical history were suggestive of ARS. Glaucoma can develop in 60%–70% of individuals with *PITX2* variants, highlighting the importance of predictive testing and monitoring for those at risk, such as her daughter [53, 88]. Additionally, *PITX2* variants are associated with systemic features, some of which may require additional clinical management [53].

Predictive genetic testing (part of what is called cascade testing) allows relatives of individuals with a genetic diagnosis to be tested to assess their risk of developing a condition. This is particularly relevant in the context of glaucoma where genetic testing can identify at‐risk relatives before they exhibit signs of the condition and lead to earlier treatment and better outcomes [82]. Individuals at risk based on genetic results can then undergo regular glaucoma monitoring to initiate treatment as soon as signs develop. Predictive genetic testing for minors is only recommended when there is a direct medical benefit during childhood [89]. Therefore, when the onset is expected to be in adulthood (e.g., *MYOC* p.Gln368Ter variant frequently associated with adult‐onset OAG), predictive genetic testing should be offered once an individual has capacity to provide informed consent for the test. However, when the onset can be expected in childhood and there is a clear benefit to treat early such as in glaucoma, predictive genetic testing and counselling can be offered and discussed with parents or caregivers (Case 2) [90].

Case 2 Cascade genetic testing and testing in minors.

A 17‐year‐old man presented to the clinic with raised IOPs (mid 20s), left central retinal vein occlusion and associated reduced visual acuity (6/6 | CF). He was diagnosed with JOAG. He had a family history of glaucoma, including his father and paternal grandfather diagnosed in their teens. At age 34, after a period of loss to follow‐up, he re‐presented with advanced field loss in the right eye and no vision in the left eye. He underwent genetic testing for MYOC based on his age at diagnosis and family history and was found to carry a pathogenic variant in MYOC associated with young age of onset. He had three children in the age range at risk of developing glaucoma. Genetic testing was offered for his children, along with genetic counselling. One of his children was identified as carrying the MYOC familial variant. They will now undergo regular monitoring for glaucoma, with a low threshold to initiate treatment should they develop signs of ocular hypertension or glaucoma.

When a genetic diagnosis is established, families can be counselled about the risk for future children and discuss potential reproductive options. This is usually more relevant to childhood or early onset glaucoma, including syndromic forms. Reproductive options that may be available to families include choosing not to have more biological children, adoption, or use of donors; prenatal genetic testing; in vitro fertilisation with preimplantation genetic testing for monogenic conditions (with or without confirmatory prenatal genetic testing); genetic testing for the child following birth or choosing to have children without undergoing any genetic testing.

### 3.3 Polygenic Testing

National Association of Testing Authorities (NATA)‐accredited PRS testing for glaucoma has been available to clinicians in Australia and New Zealand since November 2023. However, clinical guidelines to inform monitoring and management based on PRS are currently under development. The case examples described below illustrate potential scenarios on the integration of PRS into glaucoma clinical care.

#### 3.3.1 Integrated Risk Prediction for Population or Targeted Screening

**FIGURE 4:**
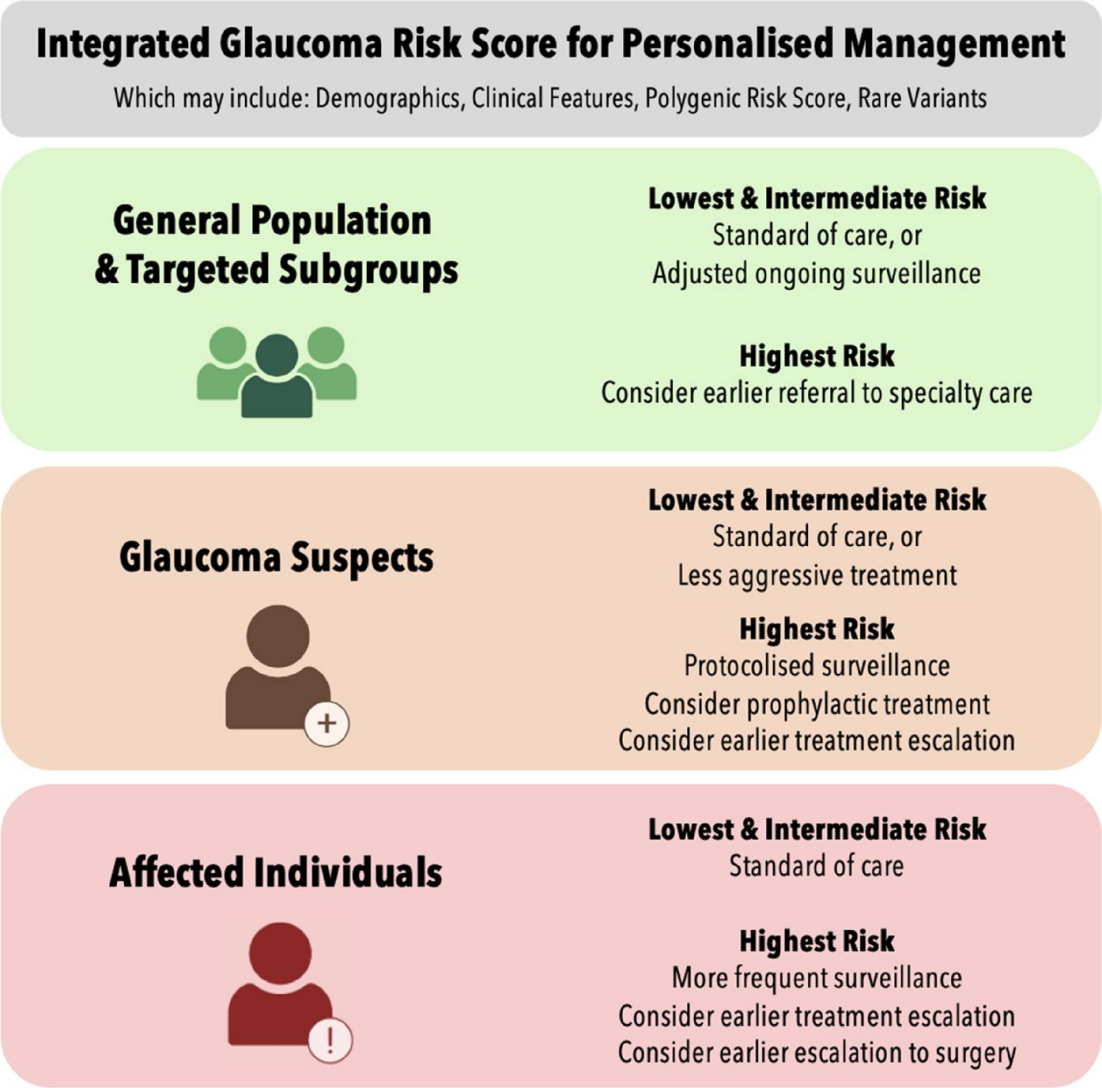
Application of an integrated genetic testing to clinical care for adult‐onset open‐angle glaucoma. Individuals are initially identified by their disease status (green: unaffected individuals within the general population or subgroups; orange: suspect cases from optometry or ophthalmology surveillance; red: affected cases from optometry or ophthalmology care). Subgroups targeted in the green box may include those with risk factors such as older age or positive family history. Personalised risk assessment could encompass an individual's demographics, any available clinical features, their polygenic risk (PRS) results and potential Mendelian test results in an integrated risk prediction tool. An ongoing monitoring schedule could then be determined by this integrated risk and disease status.

The first application arises in population screening (Figure 4, green box). The asymptomatic nature of glaucoma in the early stages, large burden of disease and effective treatment options make it ideal for screening. Current screening programmes in Australia and abroad rely on opportunistic screening but this only captures a portion of cases [83]. Previously designed screening programmes based on clinical risk factors alone were not judged to be cost effective at the time [91]. Using a glaucoma PRS for population‐based screening has been modelled to be 80% likely to be cost‐effective within the Australian and UK healthcare system [92].

Targeting screening by PRS to at‐risk groups may prove to be more cost‐effective. First‐degree relatives of affected individuals have a risk of developing glaucoma nine times higher than the general population [78, 93] and could benefit from additional risk stratification. A recent study on PRS for 24 common diseases (including glaucoma) showed a concordance rate of 33.7% of a high PRS amongst first‐degree relatives, demonstrating that a third of first‐degree relatives of an individual with a high PRS would also have a high PRS [94]. Testing relatives of high‐risk PRS individuals would enable personalised risk profiling as family members may each have different scores and could be assigned individualised monitoring schedules (Case 3). PRS and family history are independent yet provide complementary information [94]. Additional studies are needed to evaluate the variability of PRS amongst family members and the contribution of each to glaucoma risk. Ultimately, multiyear studies examining the clinical and health economic glaucoma outcomes are required to assess the appropriateness of population or targeted screening using PRS.

Case 3 Family history versus PRS to guide monitoring schedule.

Two brothers, both aged in their mid‐50s and unaffected, were undergoing routine glaucoma surveillance. They had a family history of advanced glaucoma from their father and paternal grandmother. The father's PRS was 96th percentile, and the two brothers' were 93rd and 55th percentile, associated with a 38% and 4% lifetime risk of developing glaucoma, respectively. Based on family history alone, guidelines would recommend similar surveillance schedules for both brothers. However, the PRS differentiates one sibling as higher risk than the other. The higher‐risk brother could continue surveillance with a fast‐tracked referral pathway to specialist care. The other brother's risk is similar to the general population's and his surveillance schedule could be decreased.

#### 3.3.2 Integrated Risk Prediction for Referral Triage

The second application of clinically integrated PRS testing may occur during referral triage of glaucoma suspects (Figure 4, orange box). Performing PRS testing on new referrals and integrating clinical and genetic risk factors could better inform the likelihood of glaucoma diagnosis and progression to facilitate appropriate triaging of referrals. This could reduce the waiting time for high‐risk cases and minimise the risk of irreversible field loss due to delayed clinic appointments. Furthermore, by improving triage accuracy, an integrated personalised risk score could support new care models to overcome bottlenecks in glaucoma care provision [91]. High‐risk cases could be promptly triaged and low‐risk suspects could undergo less‐frequent community‐based optometric surveillance safely (Case 4).

Case 4 Ocular hypertension with high PRS.

A 65‐year‐old man was recently referred to specialist care with elevated IOP (mid 20s) and slightly enlarged VCDR (0.6 both eyes) with normal visual fields. He had undertaken a glaucoma PRS with his optometrist, which was reported as 91st percentile. Amongst ocular hypertensives, this PRS corresponds with a 20% likelihood of treatment initiation within 3 years [68]. This referral could be triaged as more urgent than would have been otherwise with only the clinical description provided. In this case, the PRS would provide additional risk information to facilitate a timely appointment before progressive change occurs whilst awaiting specialist review.

#### 3.3.3 Integrated Risk Prediction for Personalised Monitoring Schedules

The third application of a clinically integrated PRS may arise when planning a monitoring schedule for suspect and affected individuals (Figure 4, orange and red boxes). Current guidelines recommend bi‐annual monitoring protocols or informally weighting clinical features such as IOP and field progression when deciding monitoring intervals [95]. A glaucoma PRS correlates strongly with patients requiring earlier treatment initiation and more rapid treatment escalation [68, 70]. Integrating PRS into the clinic could identify high‐risk patients requiring more frequent appointments in anticipation of treatment escalation prior to further field loss. Conversely, since low PRS is associated with less treatment initiation and escalation [68], future protocols could consider longer monitoring intervals for low‐risk individuals (Case 5).

Case 5 Glaucoma suspect with low PRS.

A 63‐year‐old man was referred to specialist care for enlarged VCDR (0.6 |0.7) with IOP in the low 20s and thick CCT (660 μm | 667 μm). He was managed conservatively with 6‐monthly monitoring and over 15 years did not display any thinning of the retinal nerve fibre layer or visual field loss. His PRS was in the 1st percentile, which amongst glaucoma suspects corresponds to a 2% likelihood of treatment initiation within 3 years [68]. Knowing this low genetic risk, this patient could have their care burden reduced through safe and appropriate reduction in appointment frequency and/or surveillance in the community setting.

#### 3.3.4 Integrated Risk Prediction for Treatment Timing

The fourth application of a clinically integrated PRS might emerge when deciding upon the timing of incisional surgery. Some patients experience progressive field loss whilst trialling less‐invasive treatment options, before ultimately requiring surgery [96]. A high glaucoma PRS has been associated with higher likelihood of escalation to incisional surgery, bilateral incisional surgery and earlier age at time to surgery [70]. Future studies should aim to develop an integrated clinical‐genetic risk threshold for patients who may benefit most from earlier surgery.

Case 6 Advanced glaucoma with high PRS.

A 67‐year‐old woman was referred to specialist care as an early manifest glaucoma with elevated IOP (30 mmHg|40 mmHg) and was commenced on topical treatment. Despite aggressive treatment escalation to maximally tolerated medical therapy and selective laser trabeculoplasty, she continued to progress (mean deviation −15 db|−13 db), requiring sequential bilateral trabeculectomy after which her visual fields stabilised. Her PRS was on the 96th percentile. Amongst affected individuals, this PRS corresponds with a 22% risk of treatment escalation within 3 years, and a 33% risk of requiring incisional surgery [68, 70]. PRS testing in this case could have led to earlier incisional surgery with less final visual field loss. Further carefully designed studies will need to explore the risk: benefit ratio and health economic implications of earlier treatment initiation, escalation and surgery in high PRS individuals with early glaucoma. Complicating this case was the patient's aversion to treatment escalation. Individuals with a high‐risk PRS were more likely to make positive behavioural changes in a recent systematic review on various common diseases [97]. Hollitt et al. previously reported that 70% of individuals with glaucoma would have had polygenic testing for glaucoma had it been available [98]. Future studies should also investigate if PRS disclosure could assist patients to better conceptualise their own risk and lead to changes in health behaviour and treatment preferences.

## 4 Ethical, Legal and Social Considerations

Genetic testing, whether for monogenic or polygenic glaucoma, necessitates careful considerations of associated implications, which include equity, informed consent and communication. Central to the implementation of any genomic test in clinical practice is equity in access. One of the main issues genomic testing is currently facing is the lack of ethnic diversity in genetic studies, which impacts both monogenic and polygenic testing. Without improved ancestral diversity, genetic testing is less likely to identify rare variants amongst under‐represented individuals and carries increased difficulty interpreting variants identified [99]. Similarly, most PRSs, including for glaucoma, has been developed in European populations [100]. Developing PRS with equivalent predictive validity across diverse ethnic groups is essential to ensure equitable access for all. Initiatives are underway to increase ancestral diversity in genomic datasets, including glaucoma [61, 101, 102]. Nevertheless, many populations such as African populations are under‐represented, which is especially relevant to glaucoma, which is more prevalent amongst populations of African ancestry [1]. Inequity considerations also encompass social determinants of health impacting access to care and testing. Sociodemographic factors including geographic location, culture and language can impact access to genetic testing [103]. These health disparities are critical to address to ensure that the potential benefits of clinical genetic testing for glaucoma can be available to all.

Patient attitudes towards genetic testing for glaucoma are positive overall. Predictive genetic testing for *MYOC* is perceived as acceptable and associated with strong perceived benefits [104, 105]. Individuals with childhood glaucoma viewed genetic testing and counselling access as important to their overall care and family planning [106]. Similarly, adults with and without established glaucoma expressed interest in PRS testing, with increased interest amongst those who perceived their risk to be high [98, 107]. Patient concerns about genetic testing (monogenic and polygenic) include cost, emotional impact of results and the potential for discrimination (employment and insurance) [104, 107].

Integrating genetic testing into the clinic leans on clinician familiarity with concepts of genetics and interpretation of results for appropriate referral, counselling and management plan. Monogenic testing is commonly managed by clinical genetic services, with the support of multidisciplinary clinics [108]. However, the scale and the range of potential applications for glaucoma polygenic testing mean that a range of healthcare professionals, including non‐genetic professionals, will be involved. Studies have reported a positive attitude of healthcare professionals towards polygenic testing for glaucoma, including amongst both genetic and ophthalmic professions [109]. Nevertheless, surveyed healthcare professionals have expressed limited knowledge and a lack of confidence in ordering or interpreting polygenic tests for glaucoma [109]. This indicates an urgent need to provide education and develop training resources [110].

Communication of PRS specifically is a novel concept requiring appropriate reporting strategies. It is imperative to convey that PRS are not diagnostic but rather provide an estimate of risk. Patients should be counselled about the potential for PRS to change over time with technical advances (via updated versions of the PRS or different providers using different algorithms), and clinicians selecting a PRS should consider its generation and accreditation status. Limitations of PRS in the context of under‐represented ancestries should be factored into the communication of results. PRS should always be interpreted in the context of other glaucoma risk factors. An individual with a low PRS could have a high risk of developing glaucoma if they have a strong family history or carry a juvenile onset pathogenic monogenic variant (e.g., in *MYOC*).

In Australia, genetic test results do not impact health insurance, which is community‐rated, but life insurance is permitted to use them (risk‐rated). Under the current self‐regulated moratorium, life insurers are prohibited to ask for or use genetic test results for life insurance cover < $AU500 000 [111]. Australians have expressed concerns about genetic discrimination by insurance companies, specifically individuals experienced difficulties accessing life insurance despite the moratorium, and some chose not to have genetic testing due to these concerns [112, 113]. On 11 September 2024, the Australian government announced a plan to legalise a total ban on the use of genetic test results in life insurance underwriting following community consultation [114]. It remains unclear at this stage what the specific impact of PRS could be on life insurance policies. A recent Australian position statement advocates for more clarity on how PRS may be used by the insurance industry [110].

A summary of common questions related to genetic testing for monogenic and polygenic glaucoma is outlined below.

**BOX 2: Common questions**

1. Does a high PRS equate to definite glaucoma?

No. PRS are risk‐stratification tools, not diagnostic tools. They can estimate the likelihood of an outcome but cannot inform diagnosis. The likelihood of glaucoma being diagnosed is also strongly dependent on age.

2. Does a monogenic variant guarantee a patient will develop glaucoma?

No. A pathogenic monogenic variant will likely mean that an individual is at an increased likelihood of developing glaucoma; however, it does not guarantee a patient will develop glaucoma.

3. Does a low PRS equate to zero glaucoma risk?

No. A low PRS indicates a very low likelihood of glaucoma due to common variants but does not eliminate the risk of glaucoma altogether. Also, polygenic testing does not currently include the sequencing of whole genes and therefore would not report on pathogenic variants associated with monogenic glaucoma such as MYOC. An individual with a low PRS may have a pathogenic monogenic variant, which could confer a significant glaucoma risk or may develop glaucoma due to other reasons (e.g., trauma, uveitis).

4. What is the role of ancestry in the PRS?

The allele frequency and its strength of association to a trait is specific to populations. Genetic variants confer differing degrees of glaucoma risk across different ancestral populations. There are different approaches to addressing ancestry in GWAS and PRS, such as including multiple ancestries in discovery GWAS, normalising a PRS to an ancestrally matched reference population and/or validating a PRS in an ancestry‐specific population. Non‐European ancestries are under‐represented in genomic databases across the globe, and increasing ancestral diversity is an urgent priority to ensure equitable access to genetic testing. This means that PRS results may currently be less predictive or not available to individuals from under‐represented ancestries or mixed ancestry. Multiple studies are currently addressing this gap by generating or validating PRS for glaucoma for major non‐European ancestries (East Asia, South Asia, African, and Hispanic).

5. Are all PRS the same?

No. PRS are built from GWAS summary statistics, of which several GWAS for open‐angle glaucoma and its endophenotypes have now been conducted.^55^ Points of differences between GWAS on glaucoma include the input trait (phenotypes and/or endophenotypes), ancestry/ies of the cohort, sample size and reference panels, all of which influence the determined final list of SNPs and their assigned strength of their association to the trait. Clear description of the derivation of the PRS and its accreditation are required for clinical use.

6. Who can benefit from monogenic testing?

There are clear benefits to monogenic testing for patients with childhood glaucoma, including to inform diagnosis, prognosis and risk to family members and/or future children. Family members of an individual with a genetic diagnosis can benefit from predictive genetic testing to establish their risk. Monogenic testing for adult‐onset glaucoma at a population level is not supported due to the low diagnostic yield and associated costs and resources required. However, clinicians can contact their local genetics service to discuss the relevance of testing if they believe it could be beneficial in certain circumstances (e.g., young age of onset associated with a strong family history, medical/family history suggesting an underlying syndrome).

7. How can I refer a patient for genetic testing?

Monogenic testing: Testing for monogenic forms of glaucoma can be coordinated alongside a clinical genetics service or research project such as referral to ANZRAG where deemed appropriate. The need for, availability and type of testing are established by the ordering clinician. Typically, clinical testing will take at least 2–3 months in most cases (testing may be able to be expedited in cases related to a current pregnancy). There is currently no Australian Medicare funding for genetic testing for isolated glaucoma in an affected individual with no family history of a known genetic variant. However, where deemed appropriate, testing may be funded by public genetics departments or hospitals. Specific criteria will vary by institution. Patients can be referred to a local clinical genetics service by a general practitioner or specialist. Referral guidelines for clinical services or research guidelines will be specific to the organisation and can be found at this URL: https://hgsa.org.au/Web/Web/HP‐Resources/Clinical‐genetics‐services‐by‐state/Clinical‐Genetic‐Services.aspx.

Polygenic testing: Genetic test results to be used in clinical decision making are required to be performed in laboratories with NATA‐accreditation in Australia. Direct‐to‐consumer tests for PRS are regulated in Australia but are available from overseas companies. However these tests may not meet the NATA standards of accreditation (or US equivalent—CLIA, Clinical Laboratory Improvement Amendments). At the time of writing, at least one glaucoma PRS had NATA accreditation (SightScore, Seonix Pty Ltd, Australia). This test is a clinician‐ordered test (not direct‐to‐consumer) available in Australia, New Zealand and the United States. Ophthalmologists and Optometrists can refer patients for PRS testing via an online portal (https://www.seonixbio.com/). The test itself is a cheek swab, which can be done in the clinic or at home. Results are returned via the portal within 6 weeks, with a report containing detailed clinical interpretation.

Research genetic testing: The ANZRAG is an international repository of genetic samples and clinical data from individuals with glaucoma and their family members. The registry recruits individuals with all types of glaucoma and severity. Gene discovery is done through exome or genome sequencing, and PRS is calculated for research purposes. Genetic testing for Mendelian glaucoma‐associated genes is performed with results validated in a NATA‐accredited laboratory and provided back to clinicians and participants by a genetic counsellor. Patients can be referred to the registry through the online portal (https://anzrag.com.au/anzrag‐referral‐form/).

## 5 Conclusions

The heritability of glaucoma and the number of effective interventions available make the condition highly suitable for genetic risk stratification. Tools integrating genetic, demographic and clinical risk factors will have the greatest potential to influence a range of scenarios. Translating the latest genetic testing applications into the clinic will offer personalised risk assessment to drive paradigm‐changing reforms and reduce not only the burden of glaucoma blindness but also the monitoring burden for lower risk glaucoma suspects.

## Conflicts of Interest

J.E.C. is coinventor of a patent (AU201890220601) and is a shareholder of Seonix Bio.

## Supporting information


Table S1.

## Data Availability

Data sharing is not applicable to this article as no new data were created or analyzed in this study.
