# Supplementary material for: Progress in Translating Glaucoma Genetics Into the Clinic: A Review
Source: Clin Exp Ophthalmol. 2025 Feb 10;53(3):246–59. doi: 10.1111/ceo.14500 (PMC11962708; doi:10.1111/ceo.14500)
Supplement: Supplementary file 1 — Table S1. [file CEO-53-246-s001.docx]

| **Supplementary Table**: List of top 100 SNPs in the Craig et al. 2020 multitrait PRS for open-angle glaucoma | | | | | | | | | | | |
| --- | --- | --- | --- | --- | --- | --- | --- | --- | --- | --- | --- |
| **rsID** | **Nearest**  **Gene** | **Consequence** | **Distance** | **MTAG**  **P-value** | **MTAG**  **OR** | **GLC**  **P-value** | **GLC**  **OR** | **IOP**  **P-value** | **IOP**  **B-coef** | **VCDR**  **P-value** | **VCDR**  **B-coef** |
| rs10918274 | TMCO1 | intronic |  | 5.55E-92 | 1.37 | 1.00E-65 | 1.38 | 9.63E-12 | 0.27 | 4.54E-01 | 1.50E-03 |
| rs2472493 | ABCA1 | non-genic | 5412 | 7.76E-59 | 1.19 | 9.00E-38 | 1.19 | 2.45E-08 | 0.14 | 5.14E-05 | 5.40E-03 |
| rs9913911 | GAS7 | intronic |  | 1.07E-58 | 1.19 | 7.00E-30 | 1.18 | 2.00E-46 | 0.22 | 2.72E-05 | 5.70E-03 |
| rs2024211 | CAV2 | non-genic | 4430 | 6.86E-49 | 0.84 | 4.46E-01 | 1.00 | 3.68E-10 | -0.18 | 5.54E-03 | -4.10E-03 |
| rs28795989 | AFAP1 | intronic |  | 5.80E-42 | 1.16 | 3.00E-25 | 1.15 | 3.00E-31 | 0.17 | 1.62E-02 | 3.30E-03 |
| rs2275241 | LMX1B | non-genic | 5631 | 3.24E-40 | 1.16 | 6.00E-19 | 1.14 | 3.65E-04 | 0.10 | 1.29E-03 | 4.40E-03 |
| rs9853115 | DGKG | non-genic | 51577 | 1.15E-39 | 1.15 | 1.00E-15 | 1.12 | 2.00E-42 | 0.20 | 5.61E-02 | 2.50E-03 |
| rs944801 | CDKN2B | non-genic | 42366 | 1.99E-38 | 0.87 | 7.00E-56 | 0.80 | 5.75E-02 | -0.05 | 5.00E-91 | -2.00E-02 |
| rs2745572 | FOXC1 | non-genic | 61781 | 1.17E-30 | 1.14 | 6.00E-21 | 1.15 | 4.00E-18 | 0.14 | 1.16E-04 | 5.30E-03 |
| rs2022945 | ANGPT1 | non-genic | 10571 | 1.11E-29 | 0.83 | 1.00E-14 | 0.85 | 5.83E-04 | -0.14 | 1.73E-02 | -4.80E-03 |
| rs1579050 | FMNL2 | intronic |  | 7.46E-26 | 0.89 | 7.00E-08 | 0.93 | 2.00E-23 | -0.15 | 7.89E-01 | -4.00E-04 |
| rs57111852 | EXOC2 | intronic |  | 6.16E-22 | 0.87 | 9.00E-13 | 0.87 | 1.05E-01 | -0.06 | 1.34E-07 | -1.02E-02 |
| rs55892100 | TES | non-genic | 39917 | 8.34E-22 | 1.11 | 3.24E-01 | 1.00 | 3.00E-19 | 0.14 | 3.54E-02 | 2.90E-03 |
| rs58073046 | ARHGEF12 | intronic |  | 1.15E-21 | 0.86 | 1.00E-14 | 0.85 | 2.16E-09 | -0.24 | 3.67E-02 | -4.20E-03 |
| rs6914444 | GMDS | intronic |  | 4.06E-21 | 1.16 | 1.00E-10 | 1.14 | 6.20E-03 | 0.10 | 9.51E-05 | 7.30E-03 |
| rs56319620 | PTPRJ | non-genic | 3189 | 4.54E-21 | 0.89 | 6.00E-06 | 0.93 | 3.05E-08 | -0.18 | 1.43E-02 | -4.00E-03 |
| rs5756813 | TRIOBP | non-genic | 2914 | 2.60E-20 | 1.11 | 5.83E-01 | 1.00 | 4.38E-04 | 0.09 | 1.43E-05 | 5.90E-03 |
| rs56251659 | LMX1B | non-genic | 45314 | 2.07E-19 | 0.91 | 1.91E-01 | 1.00 | 5.25E-02 | -0.05 | 1.24E-01 | -2.10E-03 |
| rs76325372 | ANKH | intronic |  | 4.29E-18 | 1.11 | 3.00E-10 | 1.10 | 2.57E-04 | 0.11 | 5.65E-01 | 9.00E-04 |
| rs35155027 | SIX1 | non-genic | 14965 | 6.21E-17 | 1.09 | 7.00E-33 | 1.18 | 3.14E-01 | -0.03 | 1.82E-14 | 1.03E-02 |
| rs9494457 | PDE7B | intronic |  | 6.85E-17 | 1.10 | 7.00E-10 | 1.09 | 7.00E-14 | 0.11 | 1.38E-01 | 2.00E-03 |
| rs7924522 | ETS1 | intronic |  | 6.88E-17 | 0.91 | 5.00E-11 | 0.91 | 2.00E-14 | -0.12 | 2.50E-01 | -1.60E-03 |
| rs1867409 | KBTBD8 | non-genic | 188553 | 1.06E-16 | 1.10 | 3.00E-06 | 1.07 | 5.93E-04 | 0.10 | 6.86E-03 | 3.90E-03 |
| rs11234741 | ME3 | non-genic | 18020 | 2.72E-16 | 0.90 | 1.00E-08 | 0.91 | 3.57E-04 | -0.11 | 3.71E-04 | -5.80E-03 |
| rs4102217 | SCYL1 | non-genic | 28659 | 7.76E-16 | 1.11 | 1.00E-07 | 1.10 | 1.47E-01 | 0.05 | 1.53E-12 | 1.19E-02 |
| rs8064739 | GAS7 | intronic |  | 1.19E-15 | 0.92 | 5.00E-07 | 0.93 | 1.50E-02 | -0.06 | 1.95E-01 | -1.70E-03 |
| rs11651314 | GAS7 | intronic |  | 1.47E-15 | 0.89 | * | * | 2.87E-02 | -0.08 | 5.24E-01 | -1.20E-03 |
| rs10819187 | LMX1B | non-genic | 6236 | 1.57E-15 | 0.88 | 1.16E-02 | 1.00 | 1.74E-04 | -0.15 | 1.67E-03 | -6.40E-03 |
| rs10838681 | NR1H3 | non-genic | 4483 | 1.67E-15 | 0.91 | 8.14E-01 | 1.00 | 1.16E-04 | -0.11 | 2.43E-01 | -1.70E-03 |
| rs738722 | CHEK2 | intronic |  | 2.27E-15 | 0.91 | 3.00E-09 | 0.91 | 5.02E-01 | 0.02 | 4.80E-22 | -1.43E-02 |
| rs7784849 | CTTNBP2 | non-genic | 97755 | 3.05E-15 | 0.92 | 9.67E-01 | 1.00 | 6.19E-04 | -0.09 | 3.09E-04 | -4.80E-03 |
| rs4141194 | PLEKHA7 | intronic |  | 5.20E-15 | 0.91 | 5.04E-02 | 1.00 | 3.33E-05 | -0.12 | 6.16E-01 | 7.00E-04 |
| rs17687006 | GAS7 | intronic |  | 7.63E-15 | 1.10 | 1.31E-01 | 1.00 | 4.38E-05 | 0.12 | 4.46E-01 | 1.20E-03 |
| rs9503077 | GMDS | intronic |  | 1.01E-14 | 0.92 | 5.38E-01 | 1.00 | 8.85E-04 | -0.09 | 1.20E-02 | -3.30E-03 |
| rs75265191 | ADAMTS18 | non-genic | 108913 | 1.36E-14 | 0.86 | 6.00E-06 | 0.89 | 1.15E-04 | -0.19 | 1.06E-01 | -4.00E-03 |
| rs327712 | SEMA3C | non-genic | 287690 | 1.65E-14 | 1.09 | 2.00E-07 | 1.08 | 1.53E-02 | 0.06 | 6.51E-01 | 6.00E-04 |
| rs2935072 | DLL1 | non-genic | 136518 | 2.39E-14 | 0.89 | 1.00E-09 | 0.89 | 1.71E-03 | -0.12 | 1.11E-01 | -3.10E-03 |
| rs6140009 | BMP2 | non-genic | 275279 | 2.43E-14 | 1.09 | 2.00E-09 | 1.09 | 1.51E-01 | 0.04 | 3.00E-48 | 1.00E-02 |
| rs9608740 | EMID1 | intronic |  | 2.98E-14 | 0.90 | 6.62E-01 | 1.00 | 3.45E-02 | -0.07 | 1.98E-01 | -2.20E-03 |
| rs141377403 | ANGPT1 | intronic |  | 5.62E-14 | 1.45 | 5.54E-01 | 1.00 | 2.40E-02 | 0.26 | 2.95E-01 | 6.20E-03 |
| rs4837092 | MVB12B | intronic |  | 6.59E-14 | 1.08 | 6.80E-01 | 1.00 | 2.22E-07 | 0.13 | 8.32E-01 | -3.00E-04 |
| rs12216891 | OBP2B | non-genic | 42729 | 6.73E-14 | 0.85 | 1.48E-01 | 1.00 | 3.70E-07 | -0.26 | 2.35E-04 | -9.60E-03 |
| rs17339357 | FBXO32 | non-genic | 47460 | 9.22E-14 | 0.86 | 6.00E-08 | 0.87 | 2.26E-03 | -0.16 | 8.62E-01 | -5.00E-04 |
| rs55686925 | LMX1B | intronic |  | 1.05E-13 | 0.88 | 4.99E-01 | 1.00 | 2.00E-11 | -0.17 | 8.56E-04 | -7.30E-03 |
| rs4658101 | TGFBR3 | non-genic | 68491 | 1.16E-13 | 1.10 | 9.17E-01 | 1.00 | 1.24E-01 | 0.05 | 7.00E-51 | 2.00E-02 |
| rs1579394 | BICC1 | intronic |  | 1.38E-13 | 1.08 | 4.62E-01 | 1.00 | 1.66E-02 | 0.06 | 2.15E-04 | 4.80E-03 |
| rs8009633 | FERMT2 | intronic |  | 1.47E-13 | 1.10 | 4.69E-01 | 1.00 | 9.09E-02 | 0.05 | 2.06E-01 | 2.00E-03 |
| rs2249195 | VPS13C | non-genic | 186559 | 1.62E-13 | 1.08 | 5.35E-01 | 1.00 | 3.00E-15 | 0.12 | 3.17E-02 | 2.80E-03 |
| rs66500121 | CADM2 | intronic |  | 2.58E-13 | 1.08 | 2.00E-14 | 1.11 | 2.31E-01 | 0.03 | 6.79E-01 | 6.00E-04 |
| rs2274224 | PLCE1 | missense |  | 2.73E-13 | 0.93 | 8.00E-08 | 0.93 | 5.97E-02 | -0.05 | 5.00E-10 | -5.40E-03 |
| rs150329092 | AFAP1 | intronic |  | 2.98E-13 | 0.81 | 1.19E-01 | 1.00 | 1.92E-02 | -0.19 | 5.25E-02 | -7.60E-03 |
| rs73111535 | LYPLAL1 | non-genic | 124370 | 3.39E-13 | 1.17 | 6.00E-07 | 1.15 | 2.00E-09 | 0.17 | 6.92E-01 | 1.00E-03 |
| rs3785856 | BCAS3 | intronic |  | 3.72E-13 | 0.91 | 5.00E-06 | 0.93 | 4.25E-03 | -0.09 | 6.55E-02 | -3.00E-03 |
| rs4133395 | ANGPT1 | intronic |  | 5.07E-13 | 0.93 | 9.34E-01 | 1.00 | 2.00E-11 | -0.10 | 8.11E-01 | -3.00E-04 |
| rs77064891 | ANGPT1 | intronic |  | 7.09E-13 | 1.32 | 4.09E-01 | 1.00 | 5.69E-01 | 0.06 | 6.34E-01 | 2.20E-03 |
| rs9544024 | LMO7 | intronic |  | 1.25E-12 | 0.93 | 1.00E-07 | 0.93 | 4.63E-02 | -0.05 | 6.58E-02 | -2.40E-03 |
| rs12076366 | COL24A1 | intronic |  | 1.73E-12 | 0.88 | 3.77E-01 | 1.00 | 1.75E-06 | -0.21 | 7.40E-01 | 7.00E-04 |
| rs4496939 | ANGPT1 | intronic |  | 2.13E-12 | 0.92 | 4.21E-01 | 1.00 | 1.31E-03 | -0.09 | 4.80E-01 | 1.00E-03 |
| rs12139208 | LMO4 | non-genic | 398408 | 2.15E-12 | 1.08 | 3.00E-09 | 1.09 | 1.39E-02 | 0.07 | 5.93E-02 | 2.60E-03 |
| rs4145763 | UCK2 | non-genic | 1607 | 2.48E-12 | 1.08 | 2.38E-01 | 1.00 | 4.79E-02 | 0.05 | 1.92E-01 | 1.70E-03 |
| rs78447765 | CAV2 | non-genic | 70424 | 2.82E-12 | 0.87 | 6.00E-01 | 1.00 | 5.50E-03 | -0.13 | 9.16E-02 | -4.00E-03 |
| rs324762 | TMTC2 | non-genic | 458020 | 2.85E-12 | 0.93 | 1.49E-01 | 1.00 | 5.23E-01 | -0.02 | 2.35E-20 | -1.21E-02 |
| rs10800149 | TMCO1 | non-genic | 10965 | 3.53E-12 | 1.08 | 5.39E-01 | 1.00 | 3.02E-04 | 0.09 | 8.93E-01 | 2.00E-04 |
| rs6741499 | BABAM2 | intronic |  | 3.77E-12 | 0.92 | 5.04E-01 | 1.00 | 3.93E-04 | -0.10 | 9.88E-04 | -4.90E-03 |
| rs11795066 | ANGPTL2;RALGPS1 | intronic |  | 4.99E-12 | 0.93 | 2.21E-01 | 1.00 | 1.30E-04 | -0.10 | 3.57E-01 | 1.20E-03 |
| rs4730714 | TES | non-genic | 47255 | 6.78E-12 | 0.91 | 7.81E-01 | 1.00 | 2.84E-02 | -0.08 | 4.64E-02 | -3.70E-03 |
| rs6732795 | ANTXR1 | intronic |  | 7.16E-12 | 0.93 | 6.00E-07 | 0.93 | 8.00E-10 | -0.09 | 5.64E-01 | -8.00E-04 |
| rs73148965 | TXNRD2 | intronic |  | 9.42E-12 | 1.11 | 9.00E-13 | 1.15 | 4.92E-03 | 0.10 | 1.08E-02 | 4.70E-03 |
| rs113542380 | THADA | intronic |  | 9.71E-12 | 0.87 | 6.00E-06 | 0.89 | 2.89E-03 | -0.14 | 5.72E-03 | -6.70E-03 |
| rs7951180 | FOLH1 | non-genic | 83646 | 1.03E-11 | 0.92 | 3.86E-02 | 1.00 | 3.55E-05 | -0.13 | 1.22E-01 | -2.40E-03 |
| rs722585 | GMDS | intronic |  | 1.10E-11 | 1.08 | 6.79E-01 | 1.00 | 3.28E-01 | 0.03 | 4.30E-01 | 1.10E-03 |
| rs6916701 | DLL1 | non-genic | 116924 | 1.12E-11 | 1.08 | 2.39E-01 | 1.00 | 1.56E-02 | 0.06 | 3.45E-01 | -1.30E-03 |
| rs2145826 | SUPT3H | non-genic | 30682 | 1.27E-11 | 1.07 | 1.08E-01 | 1.00 | 6.57E-01 | 0.01 | 9.07E-01 | -2.00E-04 |
| rs34952318 | JAG1 | non-genic | 522408 | 1.38E-11 | 1.18 | * | * | 8.33E-03 | 0.18 | 1.62E-01 | -5.00E-03 |
| rs34918406 | SPTSSA | non-genic | 184520 | 1.85E-11 | 0.93 | 9.62E-02 | 1.00 | 3.73E-03 | -0.08 | 1.32E-02 | -3.40E-03 |
| rs285498 | LRRC52 | non-genic | 45429 | 1.92E-11 | 0.88 | 8.59E-01 | 1.00 | 4.85E-02 | -0.09 | 8.29E-01 | 5.00E-04 |
| rs2077218 | PLCE1 | intronic |  | 2.35E-11 | 1.09 | 7.55E-01 | 1.00 | 3.10E-02 | 0.07 | 6.81E-05 | 6.20E-03 |
| rs10869665 | PCSK5 | intronic |  | 2.74E-11 | 1.08 | 5.00E-06 | 1.07 | 5.33E-03 | 0.08 | 1.63E-02 | 3.40E-03 |
| rs8142788 | ZNRF3 | intronic |  | 3.33E-11 | 0.91 | 8.35E-01 | 1.00 | 1.23E-01 | -0.05 | 3.30E-02 | -3.70E-03 |
| rs9816799 | MECOM | intronic |  | 4.46E-11 | 0.93 | 2.00E-07 | 0.93 | 1.67E-02 | -0.06 | 5.29E-02 | -2.60E-03 |
| rs1286771 | RARB | intronic |  | 4.93E-11 | 1.11 | 2.00E-06 | 1.11 | 6.33E-05 | 0.16 | 9.84E-01 | 0.00E+00 |
| rs4703855 | PTCD2 | non-genic | 29677 | 6.15E-11 | 1.08 | 8.16E-01 | 1.00 | 1.78E-01 | 0.04 | 5.34E-01 | 9.00E-04 |
| rs1874458 | CDH11 | intronic |  | 8.88E-11 | 1.07 | 4.98E-02 | 1.00 | 1.19E-04 | 0.10 | 8.56E-01 | 2.00E-04 |
| rs2526101 | THSD7A | intronic |  | 1.25E-10 | 0.93 | 4.00E-13 | 0.90 | 6.97E-02 | -0.05 | 8.73E-02 | -2.30E-03 |
| rs12713563 | MEIS1 | non-genic | 120991 | 1.79E-10 | 0.93 | 7.90E-01 | 1.00 | 1.66E-02 | -0.06 | 7.14E-01 | -5.00E-04 |
| rs73294447 | NPC2 | non-genic | 16922 | 1.86E-10 | 1.36 | 8.03E-01 | 1.00 | 4.14E-01 | 0.09 | 1.57E-01 | 7.80E-03 |
| rs11859314 | HNRNPA1L3 | non-genic | 223874 | 2.20E-10 | 0.93 | 1.98E-01 | 1.00 | 6.30E-01 | -0.01 | 3.80E-14 | -1.12E-02 |
| rs4601984 | ZNF280D | non-genic | 34823 | 2.39E-10 | 0.93 | 8.22E-01 | 1.00 | 1.38E-03 | -0.09 | 5.87E-02 | -2.60E-03 |
| rs4696856 | ABLIM2 | non-genic | 6264 | 2.49E-10 | 0.91 | 1.46E-02 | 1.00 | 2.04E-02 | -0.09 | 2.57E-01 | -2.20E-03 |
| rs4434990 | OR4C46 | non-genic | 862 | 2.77E-10 | 0.91 | 9.50E-02 | 1.00 | 2.03E-05 | -0.17 | 3.92E-01 | -1.70E-03 |
| rs440677 | KANK2 | intronic |  | 2.80E-10 | 0.93 | 6.49E-01 | 1.00 | 2.69E-02 | -0.06 | 2.77E-02 | -3.00E-03 |
| rs4420855 | LPP | intronic |  | 3.76E-10 | 1.08 | 8.00E-07 | 1.08 | 3.79E-01 | 0.03 | 3.97E-01 | -1.20E-03 |
| rs3743861 | FANCA | intronic |  | 3.98E-10 | 1.07 | 9.47E-01 | 1.00 | 1.51E-03 | 0.08 | 9.29E-01 | -1.00E-04 |
| rs74259971 | SPTBN1 | non-genic | 24260 | 4.01E-10 | 1.09 | 1.09E-02 | 1.00 | 1.40E-03 | 0.11 | 8.05E-01 | 4.00E-04 |
| rs11229165 | OR4A16 | non-genic | 980 | 4.13E-10 | 0.91 | 1.00E-01 | 1.00 | 4.44E-05 | -0.16 | 5.86E-01 | -1.10E-03 |
| rs2226566 | ME3 | intronic |  | 5.14E-10 | 0.92 | 7.09E-01 | 1.00 | 8.58E-03 | -0.09 | 7.10E-02 | -3.00E-03 |
| rs12045227 | ST7L | intronic |  | 5.27E-10 | 0.92 | 1.74E-01 | 1.00 | 1.87E-02 | -0.08 | 8.39E-02 | -2.80E-03 |
| rs56152426 | COL4A1 | intronic |  | 5.65E-10 | 1.09 | 3.00E-06 | 1.09 | 3.28E-03 | 0.10 | 4.55E-03 | 5.00E-03 |
| rs28689523 | AFAP1 | intronic |  | 6.10E-10 | 1.07 | 6.21E-02 | 1.00 | 5.59E-03 | 0.07 | 8.72E-01 | -2.00E-04 |
| rs1755056 | RUNX2 | non-genic | 3841 | 7.42E-10 | 1.07 | 1.60E-01 | 1.00 | 1.00E-09 | 0.09 | 3.90E-01 | -1.20E-03 |
| The top 100 SNPs in the multi-trait glaucoma PRS by Craig et al. 2020. SNPs are ordered by the strongest level of association (p-value) from the multi-trait genome-wide association study (MTAG)^30^ which leverages the correlation of 3 input traits (glaucoma^30^, IOP^63^ and VCDR^30^). The odds ratios (OR) for glaucoma diagnosis from this MTAG study are reported alongside. The level of association and OR/beta-coefficient for each SNP from the separate genome-wide association studies (GWAS) for the individual input traits (glaucoma, IOP and VCDR) are also listed in corresponding columns. SNPs with a P-value approximately 1.0 and OR approximately 0.0 are denomated with an asterix (*).    rsID, unique identifier of the single nucleotide polymorphism (SNP) as per Human Genome Reference hg19; Distance, the number of base pairs the SNP from the nearest gene; MTAG, multi-trait analysis of GWAS; GLC, glaucoma; IOP, intraocular pressure; VCDR, vertical cup:disc ratio; P-value, level of significance in genome-wide association study (GWAS); OR, odds ratio derived from GWAS, where >1.00 equals greater risk towards the binary outcome and <1.00 is protective against the binary outcome; B-coef, beta-coefficient derived from GWAS, where a positive value is associated with a higher trait value for the continuous variable. | | | | | | | | | | | |
